# Severe Hepatotoxicity From Capmatinib: A Case Report and Therapeutic Approach

**DOI:** 10.7759/cureus.77652

**Published:** 2025-01-19

**Authors:** Mina Aiad, Harsh J Bhalala, Halle Bagshaw, Sophia Starner, Debasmita Saha

**Affiliations:** 1 Internal Medicine, St. Luke's University Health Network, Bethlehem, USA; 2 Internal Medicine, St. Luke's University Hospital, Bethlehem, USA; 3 Internal Medicine, The Temple/St. Luke's School of Medicine, Bethlehem, USA; 4 Hematology and Medical Oncology, St. Luke's University Health Network, Bethlehem, USA

**Keywords:** capmatinib, drug-induced liver injury, kinase inhibitor, metastatic non-small cell lung cancer, met tyrosine kinase inhibitors, n-acetyl cysteine (nac), non-small cell lung carcinoma (nsclc), transaminitis-raised liver enzyme, ursodeoxycholic acid

## Abstract

Capmatinib, a selective mesenchymal-epithelial transition (MET)-kinase inhibitor, is approved for treating metastatic non-small cell lung cancer (NSCLC) with MET exon 14 (METex14) skipping mutation. Although a known side effect, not much data is available on the management of capmatinib-induced liver injury. Here, we present a case of a 60-year-old male with MET exon mutated NSCLC who developed grade 4 liver injury after capmatinib initiation, which did not respond to drug discontinuation and eventually responded to N-acetyl cysteine (NAC) and ursodeoxycholic acid (ursodiol) therapy. This case demonstrates that NAC plus ursodiol can be an effective treatment strategy in such patients.

## Introduction

Capmatinib is an orally available kinase inhibitor that targets the mesenchymal-epithelial transition factor (c-MET), also known as the hepatocyte growth factor receptor (HGFR). C-MET aberrations via mutations, overexpression, or amplification are found in many types of cancers and lead to activating signaling pathways that promote tumor growth and metastasis [1]. On August 10, 2022, the Food and Drug Administration (FDA) granted regular approval to capmatinib (brand name: Tabrecta) for the treatment of metastatic non-small cell lung cancer (NSCLC) with MET exon 14 (METex14) skipping mutation. 

The safety of capmatinib was evaluated in the GEOMETRY mono-1 study, in which 373 patients received 400 mg orally twice daily until disease progression or unacceptable toxicity. Serious adverse reactions occurred in 53% of the patients. Elevation of alanine transaminase/aspartate transaminase (ALT/AST) occurred in 14% of the patients, and about half of them (7%) had grade 3 or 4 transaminitis, defined as >5 and >20 times the upper normal limit, respectively [2]. The median time to onset of grade 3 or higher ALT/AST elevation was 1.8 months, ranging from 0.5 to 46.4 months [3]. 

The FDA’s FDALabel online database provides instructions on dose modification, including holding or permanently discontinuing capmatinib in the case of grade 3 or 4 ALT/AST elevation, with or without bilirubin elevation. However, information on managing capmatinib-induced hepatotoxicity is scarce. 

Here, we present a case of grade 4 capmatinib-induced hepatotoxicity that did not improve with the discontinuation of the drug. Significant improvement was noted as early as one day after the administration of N-acetyl cysteine (NAC) and ursodeoxycholic acid (ursodiol), with near resolution of hepatotoxicity in two months. More case reports and studies on target therapies and possible adverse effects are important because their use is increasing, and some of their adverse effects, which we still learn about, could be life-threatening.

## Case presentation

A 60-year-old male with type 2 diabetes and limited tobacco history but no other toxic exposure was initially admitted to the hospital with left thigh pain.  Workup demonstrated a metastatic lesion in the left femoral shaft, with marked cortical thinning and right iliac chain lymphadenopathy suggesting metastatic disease. Imaging of the chest and abdomen showed a large right hilar mass, concerning for primary bronchogenic malignancy, as well as a right adrenal nodule suspicious for metastatic disease. He underwent left femur prophylactic intramedullary nail insertion, and a bone biopsy confirmed metastatic carcinoma with squamous cell and adenocarcinoma patterns.

**Figure 1:**
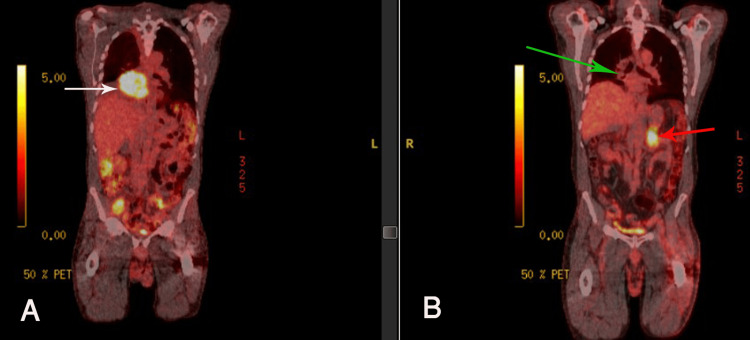
NM PET-CT before (A) and after (B) treatment with carboplatin, paclitaxel, and atezolizumab. Image (A) is remarkable for FDG-avid right hilar mass (white arrow) measuring 8.5 x 6.8 x 6.3 cm with SUV max of 11.3. Image (B) shows decreasing patchy consolidation in the right infrahilar region (green arrow) without significant focal uptake, SUV max of 1.9, previously 2.5, and new FDG avid left adrenal mass (red arrow) suspicious for metastasis. NM PET-CT: nuclear medicine positron emission tomography-computed tomography; FDG: F-18 fluorodeoxyglucose

The patient completed six cycles of carboplatin, paclitaxel, and atezolizumab, followed by 10 cycles of maintenance atezolizumab monotherapy without immune-mediated toxicity. A follow-up positron emission tomography-computed tomography (PET-CT) showed a response, but a new left adrenal mass was suspicious of metastasis (Figure 1). 

**Figure 2:**
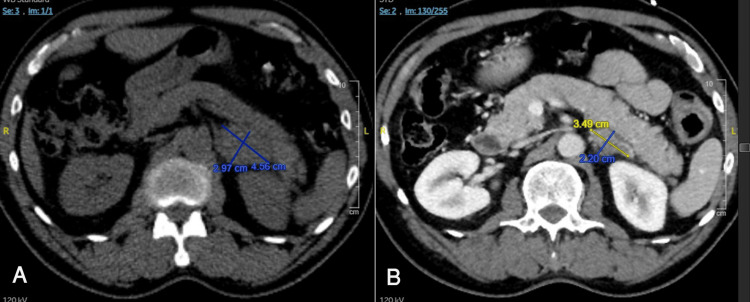
CT scan before (A) and two months after (B) treatment with capmatinib. Left adrenal mass size decreased from 4.56 x 2.97 cm to 3.49 x 2.20 cm. CT: computed tomography

The previous left femur tissue specimen was sent for molecular testing, which showed a PDL-1 (22c3) tumor proportion score of 50%, high tumor mutation burden (11 mutation/megabase), and METex14 skipping mutation, suggesting the benefit of using MET inhibitors, e.g., capmatinib or tepotinib. The treatment was then switched to the c-Met inhibitor oral capmatinib 400 mg twice daily. The patient initially tolerated the capmatinib, and a repeated CT scan showed decreased left adrenal mass size after two months (Figure 2).

However, starting week 4, there was a gradual rise in the ALT and AST. At week 8 of capmatinib, the patient had CTCAE grade 4 hepatic toxicity as evident by both AST and ALT greater than 20 times the upper limits of normal. Capmatinib was held for two weeks, but still with significantly elevated transaminases, worsening alkaline phosphatase, and bilirubin levels (Table 1). The hyperbilirubinemia was predominantly conjugated bilirubin, i.e., 11.87 mg/dL. The patient presented to the hospital with nausea, jaundice, and pruritus over the past three weeks. 

**Table 1:** Trend of liver enzymes before the initiation of NAC and ursodiol. AST: aspartate aminotransferase; ALT: alanine transaminase; ALP: alkaline phosphatase; NAC: N-acetyl cysteine, U/L: units per liter; mg/dL: milligrams per deciliter

Capmatinib	Week 0	Week 1	Week 4	Week 6	Week 8	Week 10
AST (13-39 U/L)	17	12	50	167	855	794
ALT (7-52 U/L)	12	15	84	260	1,773	1,721
ALP (34-104 U/L)	98	113	96	120	170	440
Total bilirubin (0.20-1.00 mg/dL)	0.29	0.40	0.73	0.87	1.60	19.10

The right-upper-quadrant ultrasound and MRI with MRCP were unremarkable. Laboratory testing ruled out an elevated ammonia or acetaminophen level. Testing for hepatitis B or C, herpes simplex virus 1 or 2, and cytomegalovirus was all negative. Based on the clinical scenario and the lack of any other potential causes, a diagnosis of capmatinib-induced liver toxicity was established, and a liver biopsy was deferred. 

We identified two published case reports with similar severe liver toxicity secondary to capmatinib, who were treated with hepatoprotective and anticholestatic drugs with good response [4]. 

Our patient was treated with intravenous NAC and ursodeoxycholic acid (Ursodiol). NAC was given at 150 mg/kg over one hour, followed immediately by 50 mg/kg over four hours, then 100 mg/kg administered over 16 hours, and ursodiol 4 mg/kg divided into two doses daily for 30 days. Systemic steroids were not used.

There was a significant response to the NAC and ursodiol, with AST, ALT, ALP, and T. Bilirubin gradually trending down to near-normal values over eight weeks (Table 2). In parallel, the patient's nausea, itching, and jaundice improved and mostly resolved within three days of starting the treatment.

**Table 2:** Trend of liver enzymes after initiation of NAC and ursodiol. AST: aspartate aminotransferase; ALT: alanine transaminase; ALP: alkaline phosphatase; NAC: N-acetyl cysteine, U/L: units per liter; mg/dL: milligrams per deciliter

NAC + ursodiol	Day 0	Day 1	Day 2	Day 3	Week 2	Week 4	Week 6	Week 8
AST (13-39 U/L)	794	633	616	622	237	15	13	12
ALT (7-52 U/L)	1,721	1,282	1,202	1,146	665	28	10	10
ALP (34-104 U/L)	440	335	329	294	378	174	118	146
Total bilirubin (0.20-1.00 mg/dL)	19.10	16.53	13.95	11.93	6.17	2.77	1.29	0.92

## Discussion

This case report highlights a rare but significant side effect of acute liver injury caused by the MET inhibitor, capmatinib. While the full mechanism by which capmatinib causes liver toxicity is not fully understood, previous studies suggest possible theories and explanations. C-MET is a receptor tyrosine kinase found in many normal cells, including hepatocytes. When activated via binding to its ligand hepatocyte growth factor (HGF), the HGF/c-MET signaling pathway plays a critical role in tissue regeneration and tumor mitogenesis, motility, invasiveness, morphogenesis, and angiogenesis. C-MET mutations, overexpression, and amplification are found in several cancers, including liver, renal, colorectal, and NSCLC.

Disruption of the c-MET signaling in mice liver cells made hepatocytes more prone to Fas-induced apoptosis [5]. Capmatinib 5 mg/kg reduced diethylnitrosamine (DEN)-induced liver injury in mice and inhibited liver infiltration by inflammatory cells. However, at a higher dose of 10 mg/kg, it aggravated DEN-induced hepatocellular ballooning and apoptosis [6]. 

Moreover, capmatinib is metabolized in the liver via the cytochrome P450 system, primarily by CYP 3A4 and aldehyde oxidase. Therefore, it has drug interaction with agents that can inhibit or induce these enzymes [7]. Our patient was on metformin 1000 mg twice daily for his type 2 diabetes, which he continued along with the capmatinib. Metformin is known to suppress the expression of CYPA3A4 in hepatocytes [8], therefore increasing the plasma level of capmatinib. 

Another suspected risk factor for capmatinib-induced liver toxicity is previous treatment with immunotherapy. The reported outcomes of the phase 2 GEOMETRY mono-1 study show that 16% of the previously treated patients, compared to 11.6% of the treatment-naive patients, developed ALT elevation on capmatinib. CTCAE grade 4 liver toxicity was also more common in those previously exposed to immunotherapy [9]. When capmatinib was given with pembrolizumab for patients with NSCLC PDL1 TPS ≥50%, the combination was not tolerable; the most common adverse effect was ALT and AST elevation. Grade 3 or higher AST and ALT elevation occurred in about 10% of patients taking capmatinib plus pembrolizumab vs. only 4% of patients on monotherapy pembrolizumab [10]. 

According to the FDA’s FDALabel online database, the median time to onset of grade ≥3 hepatotoxicity was 1.4 months (0.5 to 4.1 months). The current recommendations include monitoring liver function tests before starting capmatinib, every two weeks during the first three months, and then monthly after or sooner if clinically indicated. If AST or/and ALT elevation is without concomitant bilirubin elevation or if bilirubin is elevated without AST/ALT elevation, the recommendation is to hold capmatinib. If these elevated labs normalize within seven days, capmatinib will be resumed at the previous dose. Still, if it takes more than seven days to normalize, the recommendation is to decrease the dose. In the case of AST or/and ALT elevation with bilirubin elevation (unless it has a different etiology, e.g., hemolysis or cholestasis), capmatinib will be discontinued [11]. 

Our patient had grade 4 hepatic toxicity with AST, ALT, and bilirubin elevation eight weeks after initiating capmatinib, in the absence of any evidence for hemolysis or cholestasis. Discontinuing the capmatinib in his case was not enough for the liver enzymes to improve, but total bilirubin increased significantly two weeks after the drug discontinuation. NAC, an antioxidant with hepatoprotective properties, and ursodeoxycholic acid, known for its cytoprotective effects by alterations in the bile acid pool, were chosen based on their easy availability, documented efficacy, and safety in cases of another drug-induced hepatotoxicity and in instances of prior capmatinib-induced liver injury [4]. The significant improvement in liver function tests within days of initiating treatment and normalization of these tests over eight weeks reinforces the utility of these therapies in managing capmatinib-induced liver injury. 

Ademetionine (S-adenosyl-L-methionine; SAMe) is another agent used in various liver diseases to maintain glutathione levels and reduce hepatocyte damage. While SAMe was used in the previous cases (intravenous 500 mg daily) in addition to the NAC and ursodiol, our patient’s hepatotoxicity was managed successfully with NAC plus ursodiol alone. Notably, patients in those three cases, including ours, had significant improvement without administering corticosteroids; hence, it suggests that corticosteroids should be avoided to decrease complication risk. 

## Conclusions

Capmatinib, a selective MET-kinase inhibitor, is approved for treating metastatic NSCLC with METex14 skipping mutation. Capmatinib-induced liver injury was reported in about 14% of the patients, with 7-8% having severe liver injury (CTCAE grade 3 or higher). Risk factors include prior or concurrent use of immunotherapy and being on CYPA3A4 inhibitor(s). The first step in managing severe capmatinib-induced liver toxicity is holding the agent. The optimal next steps in managing persistently elevated liver enzymes despite holding off the capmatinib are still unknown. To the best of our knowledge, here, we present the third case reporting the efficacy of NAC plus ursodeoxycholic acid in managing such severe cases. We propose that adding ademetionine (SAMe) may not be necessary, and similarly, steroids could be avoided with still great response and fewer adverse effects. Further research is warranted to validate these findings and to explore additional strategies for managing capmatinib-induced hepatotoxicity, especially when severe and life-threatening.
